# Plasma Adiponectin and Its Correlation with Carotid Intima-Media Thickness in Obesity and in Type 2 Diabetes and Nonalcoholic Fatty Liver Disease

**DOI:** 10.1155/2023/6661585

**Published:** 2023-08-31

**Authors:** Maha Hussein, Aasem Saif, Mona Amin, Osama Khalafallah, Ahmed Hussien, Samar Aboulsoud, Shrook Mousa

**Affiliations:** ^1^Internal Medicine Department, Cairo University, Giza, Egypt; ^2^Clinical and Chemical Pathology Department, Cairo University, Giza, Egypt

## Abstract

**Methods and Results:**

The study included 200 Egyptian subjects. They were divided into four equal groups: group 1: obese patients with NAFLD and T2DM (O+/NAFLD+/DM+), group 2: nonobese patients with NAFLD and T2DM (O-/NAFLD+/DM+), group 3: obese nondiabetic patients with NAFLD (O+/NAFLD+/DM-), and group 4: nonobese healthy control subjects. Plasma adiponectin was measured using ELISA (enzyme-linked immunosorbent assay) technique. Ultrasonography was used to diagnose NAFLD. CIMT was assessed using Doppler ultrasonography. Plasma adiponectin was significantly lower and CIMT was significantly higher in O+/NAFLD+/DM+, as compared with O-/NAFLD+/DM+, O+/NAFLD+/DM-, and control subjects (*p* < 0.001 for all). A significant negative correlation was found between adiponectin and CIMT in obese patients with NAFLD (*p* < 0.05), but not in patients with NAFLD and T2DM. The significant independent predictors of CIMT were diabetes duration, BMI (body mass index), albumin/creatinine ratio, and cholesterol.

**Conclusion:**

Plasma adiponectin is inversely correlated with CIMT in obese patients with NAFLD, but not in patients with NAFLD and T2DM. Hypoadiponectinemia could be a good indicator of cardiovascular risk in obese patients with NAFLD, with or without T2DM, but not in nonobese patients with NAFLD.

## 1. Introduction

Adiponectin is one of the adipokines that is secreted and produced by adipose tissues. It is well known for its benefits and role in cardioprotection, as it has antiatherogenic, anti-inflammatory, and antidiabetic effects [1–3]. In obese individuals, adiponectin plasma levels and expression are decreased [4, 5]. Also, patients with nonalcoholic fatty liver disease (NAFLD) have low plasma levels of adiponectin [6].

Increasing BMI (body mass index) and visceral obesity are associated with decreased level of circulating adiponectin. When weight loss occurs, circulating level of plasma adiponectin increases; this means that in obesity the decrease level of adiponectin is reversible [7]. On the contrary, it was found that the circulating level of adiponectin is increased in patients with liver cirrhosis regardless of body mass index, age, or even disease etiology. There has been inverse correlation between adiponectin levels and the degree of fibrosis and steatohepatitis [8]. In patients with type 2 diabetes, carotid intima-media thickness (CIMT), as one of the markers of atherosclerosis, was negatively correlated with plasma adiponectin level [9]. It has been reported that obesity might partially explain this inverse relationship [10].

## 2. Pleiotropic Effects of Adiponectin

Adiponectin has an important role in the metabolism of the liver and skeletal muscle [2].

Adiponectin affects the energy metabolism as it regulates the fat lipid metabolism by inhibiting lipolysis [11]. It exhibits a protective role in many inflammatory diseases such as insulin resistance, cardiovascular diseases, and atherosclerosis. It decreases the inflammation in macrophages, epithelial cells, and endothelial muscle [12].

Moreover, it can cause cell growth inhibition and apoptosis induction through different molecular pathways. It is involved in different cancer types as breast, endometrial, stomach, and leukemia [13]. The dysregulated production of the adipokines and the cytokines is strongly correlated with the occurrence of the complications of the obesity including cardiovascular diseases, diabetic retinopathy, metabolic syndrome, respiratory disorders, and cancer [14].

The aim of the work was to evaluate the plasma level of adiponectin and its correlation with CIMT in Egyptian patients with NAFLD and to study the effects of obesity and T2DM on the correlation between CIMT and adiponectin.

## 3. Methods

The study included 200 Egyptian subjects (103 females). Their ages ranged between 44 and 63 years whom visited the internal medicine and diabetes clinics at the Cairo University hospital. Our study participants were divided into four groups (each included 50 subjects): group 1: obese (BMI more than 30 kg/m^2^) patients with NAFLD and T2DM >5 years (O+/NAFLD+/DM+), group 2: nonobese (BMI less than 25 kg/m^2^) patients with NAFLD and T2DM >5 years (O-/. Bhv NAFLD+/DM+), group 3: obese (BMI ≥ 30 kg/m^2^) nondiabetic patients with NAFLD (O+/NAFLD+/DM-), and group 4: nonobese (BMI < 25 kg/m^2^) normal healthy control subjects. Participants were selected to reflect the local demographic with all groups deliberately matched for age and sex. Concerning the medications used by O+/NAFLD+/DM+ and O-/NAFLD+/DM+, there were no differences.

We did hepatitis C and B viral markers to our patients and they were all negative. Patients with history of alcohol intake or hepatitis, high blood pressure, impaired renal function, atherosclerotic cardiovascular disease, pregnant women, and those with possible primary hyperlipidemia (total cholesterol >300 mg/dL or LDL cholesterol >190 mg/dl or triglycerides >300 mg/dl with history of xanthomas or family history of myocardial infarction before 55 years of age) were excluded from our study. The participants were provided with written formal consents. The study protocols and the process for obtaining informed consent were approved by the Cairo University ethical committee and review board.

All participants were subjected to a complete physical examination including body mass index (BMI) assessment and blood pressure (BP). Laboratory work up included serum fasting and postprandial blood glucose (FBG and PPBG), glycated hemoglobin (HbA1c), lipid profile, kidney function tests, and urinary albumin/creatinine ratio (ACR). Plasma adiponectin level was measured using an ELISA kit (Millipore, St. Charles, Missouri, USA).

All participants were examined by abdominal ultrasound to assess the liver by using a convex probe 3.5 MHz. The ultrasound was done by the same operator using the same machine (ALT HDI Ultramark). Four ultrasound findings were used to diagnose NAFLD which were the presence of the hepatorenal contrast, liver brightness, deep attenuation, and blurring of the vasculature. In addition to the ultrasonographic findings, all participants have negative viral markers for hepatitis B and C with no history of alcohol consumption or medications or any other liver disease that can cause fatty liver [15].

We used color-coded Doppler ultrasonography with high resolution to measure CIMT. All participants were examined by the same operator using the same machine (ALT HDI, Ultramark) using a B-mode and 12 MHz linear probe. The participants were in the supine position, with the head turned 45° from the side during scanning. We took the beginning of the dilatation of the carotid bulb, with loss of the parallel configuration of the near and far walls of the common carotid artery, as our reference point for the measurement of CIMT. The operator located the leading edges which correspond to the transition zones between lumen-intima and media-adventitia over a length of one centimeter proximal to the reference point at its thickest point, not including plaques. Plaque was identified as a localized thickened lesion (≥1.1 mm). All the results were expressed as the mean of both sides. The mean CIMT of four measurements were recorded for each patient.

Data were analyzed by the Statistical Package of Social Science, Software program, version 23 (IBM Corp. Released 2015. IBM SPSS Statistics for Windows, Version 23.0. Armonk, NY: IBM Corp.). Data were presented as mean ± standard deviation (SD), to compare between groups, we used the ANOVA test with the post hoc Tukey test (if parametric) or Kruskal–Wallis test followed by Mann–Whitney test (if nonparametric) for quantitative variables and the chi square and Fisher's exact test for qualitative ones. Spearman correlation coefficients (*r* values) were calculated to signify the association between different quantitative variables. Regression analysis was used to evaluate the independent association of risk factors. *P* < 0.05 was considered statistically significant.

## 4. Results

The demographic and laboratory data of the four groups are shown in Table 1.

Plasma adiponectin was significantly lower in patients with NAFLD as compared with control subjects (*p*  < 0.001). Adiponectin was significantly lower in O+/NAFLD+/DM+ (group 1) as compared with O-/NAFLD+/DM+ (group 2), O+/NAFLD+/DM- (group 3), and control subjects (group 4) (15.2 ± 3.1 vs 26.3 ± 12.1, 37.6 ± 20.5, and 47 ± 11.5 *µ*g/dl, respectively, *p*  < 0.001) (Table 1).

**Figure 1:**
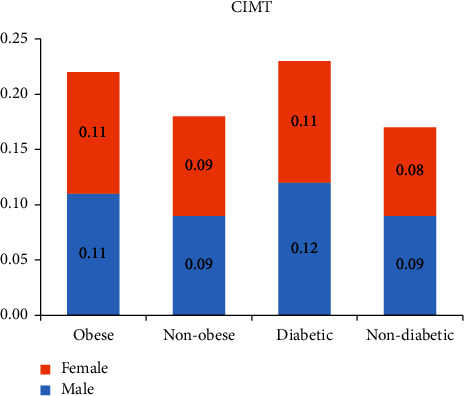
Mean value of carotid intima-media thickness in obese, nonobese, diabetic, and nondiabetic female and male patients.

Adiponectin was also significantly lower in obese compared to nonobese patients (*p*  < 0.001) and in patients with T2DM compared to nondiabetic patients (*p*  < 0.001) (Table 2 and Figure 1).

There was a significant negative correlation between plasma adiponectin and age, triglycerides, LDL (*p* < 0.05 for all), cholesterol (*p* < 0.01), CIMT, BMI, FBG, PPBG, HbA1c, and urinary ACR (*p*  < 0.001 for all) in patients with NAFLD (Table 3).

CIMT was significantly higher in patients with NAFLD as compared with control subjects (*p*  < 0.001). CIMT was significantly higher in O+/NAFLD+/DM+ (group 1) as compared with O-/NAFLD+/DM+ (group 2), O+/NAFLD+/DM-/ (group 3), and control subjects (group 4) (0.12 ± 0.03 vs. 0.11 ± 0.03, 0.10 ± 0.02, and 0.07 ± 0.01 mm, respectively, *p*  < 0.001) (Table 1).

**Figure 2:**
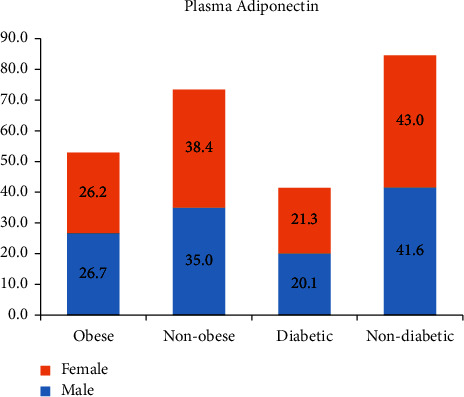
Mean value of plasma adiponectin in obese, nonobese, diabetic, and nondiabetic female and male patients.

CIMT was significantly higher in obese compared to nonobese subjects (*p*  < 0.001) and in patients with T2DM compared to nondiabetic patients (*p*  < 0.001) (Table 2 and Figure 2).

There was a significant positive correlation between CIMT and age (*p* < 0.05), diabetes duration (*p* < 0.01), BMI, FBG, PPBG, HbA1c, urinary ACR, cholesterol, TG, and LDL (*p* <  0.001 for all) in patients with NAFLD. No significant difference was found between males and females in any of the studied parameters (Table 3).

A significant negative correlation was found between plasma adiponectin and CIMT in patients with NAFLD (*p* < 0.05). A significant inverse correlation was found between plasma adiponectin and CIMT in obese patients with NAFLD (*p* < 0.05), but no significant inverse correlation could be established between plasma adiponectin and CIMT in patients with T2DM (Table 4).

Backward stepwise linear regression model was used to explore the predictors of CIMT, and variables entered step 1 were age, diabetes duration, BMI, fasting glucose, 2-hour postprandial blood glucose, HbA1c, creatinine, albumin/creatinine ratio, cholesterol, triglycerides, LDL, HDL, and plasma adiponectin. The last step revealed that the significant (independent) predictors of CIMT were only diabetes duration, BMI, albumin/creatinine ratio, and cholesterol (Table 5).

## 5. Discussion

We assessed plasma adiponectin and its correlation with CIMT, which is used as a marker of atherosclerosis, in Egyptian patients with NAFLD. We studied the effects of obesity and T2DM on the correlation between adiponectin and CIMT. Our results showed that plasma adiponectin level was significantly lower in O+/NAFLD+/DM+ as compared to O-/NAFLD+/DM+, O+/NAFLD+/DM-, and control subjects. Plasma adiponectin was significantly lower in obese as compared with nonobese subjects in the whole study population with a *p* value <0.00.

Adiponectin plays an important role concerning energy metabolism. Its plasma level is decreased in obesity and increases after weight loss [5, 16, 17]. In T2DM and obesity changes in the expression of adiponectin or adiponectin receptors decreases insulin sensitivity which leads to insulin resistance which in turn cause aggravation of the hyperinsulinemia. When weight loss occurs, there is rise in adiponectin levels with a specific increase in the most biologically active oligomers [18].

Assal et al. reported that plasma adiponectin was significantly lower in obese patients with T2DM compared to obese nondiabetic and nonobese healthy subjects, but they did not include a group of nonobese patients with T2DM or patients with NAFLD in their study [19]. Both Nayak et al. and Hara et al. reported that regardless of the diabetic status, plasma adiponectin levels were reduced in obese compared to nonobese individuals [20, 21].

In this study, plasma adiponectin was significantly lower in patients with T2DM as compared with nondiabetic patients. Fifty to seventy percent of patients with diabetes have NAFLD, and its severity is worsened by the presence of diabetes [22].

These results agree with those of Aleidi et al. who reported lower plasma adiponectin levels in patients with T2DM as compared with healthy subjects. But their study group was much smaller, including only 80 subjects (61 patients with T2DM and 19 healthy control subjects). They also reported that obese females with T2DM had lower adiponectin levels than nonobese females with T2DM, but they did not find similar results in male patients with T2DM [23].

Plasma adiponectin levels were significantly lower in patients with NAFLD as compared with normal healthy control subjects. In NAFLD, reduced levels of plasma adiponectin are related to the amount of hepatic fat content and hepatic insulin sensitivity. In NAFLD, hypoadiponectinemia is thought to be part of a metabolic disturbance which is characterized by the accumulation of ectopic fat in the central compartment [6].

This is in agreement with those of Bugianesi et al. who reported significantly lower plasma adiponectin levels in patients with NAFLD as compared with healthy control subjects in Italy, but their study participants were mainly males [6]. Fadaei et al. also reported a lower adiponectin level in Iranian patients with NAFLD as compared with a control group [24].

In this study, there was a significant negative correlation between plasma adiponectin and BMI, FBG, PPBG, HbA1c, urine ACR, cholesterol, triglycerides, and LDL in patients with NAFLD, with or without diabetes. These findings were consistent with those of Ahn et al., Goropashnaya et al., and Narayan et al. [25–27]. Mantzoros et al., Ljubic et al., and Jung et al. [28–30]. Salman et al. [31] and Bugianesi et al. reported an inverse relation between plasma adiponectin and BMI in patients with NAFLD [6].

A study by Cai et al. reported that when adiponectin was overexpressed in atherosclerotic ApoE^−/−^ mice, it significantly decreased the area and size of carotid atherosclerotic lesions. This was done through preserving NO from destruction and inhibiting superoxide production These are the major mechanisms by which adiponectin exerts its vascular protective and antiatherosclerotic and effects. iNOS expression is inhibited by the adiponectin. Therefore, its antinitrative and antioxidative effects reduce atherosclerosis [32].

CIMT has been reported to be a representative of subclinical and asymptomatic atherosclerotic vascular diseases. It correlates with the extent of atherosclerotic lesions elsewhere in the body [33–35]. In our study, CIMT was significantly higher in O+/NAFLD+/DM+ , as compared to O-/NAFLD+/DM+, O+/NAFLD+/DM-/, and control subjects. It was significantly higher in obese patients compared to nonobese ones, and in patients with T2DM compared to nondiabetic ones and in patients with NAFLD as compared with control subjects. These results agree with those of Dalmas et al. who reported that CIMT was higher in obese patients with T2DM as compared with obese nondiabetic and nonobese subjects; however, they did not include a group of nonobese patients with T2DM or patients with NAFLD in their study [36]. Both Pillai et al. and Kota et al. reported a higher CIMT in patients with diabetes as compared with nondiabetic individuals [37, 38]. Vasilescu et al. found a higher CIMT in obese patients with newly diagnosed T2DM as compared with obese nondiabetic subjects [39]. On the other hand, Kowall et al. found no significant increase in CIMT among patients with diabetes or prediabetes, as compared with nondiabetic subjects, after adjustment for sex, age, and anthropometric variables [40]. Fracanzani et al. reported a significant increase in CIMT in patients with NAFLD, as compared with normal control subjects. They also reported a significant positive correlation between CIMT and total and LDL cholesterol in those patients [41].

We found a significant positive correlation between CIMT and age, diabetes duration, BMI, FBG, 2HPPG, HbA1c, urine ACR, cholesterol, triglycerides, and LDL in patients with NAFLD, with or without diabetes. Similar correlations were recorded by Diggikar et al. and Park et al. [42, 43]. Eric et al. reported a statistically significant correlation between CIMT and BMI in male, but not in female, Nigerian adults with primary hypertension [44]. Gao et al. reported an association between CIMT and FBG among low‐income adults with prediabetes and diabetes in rural China [45]. A study done on Asian Indians by Venkataraman et al. found a significant increase in CIMT with increasing levels of HbA1c even among subjects with normal glucose tolerance [46]. In an Iranian study, Shahrokh et al. found a significant relationship between CIMT and renal parameters including albuminuria and estimated glomerular filtration rate in patients with T2DM [47]. Similar results were reported in India by Gayathri et al. [48]. Fracanzani et al. reported a significant positive correlation between CIMT and total and LDL cholesterol in patients with NAFLD [41].

Alizargar and Bai reported that age, waist circumference, systolic BP, and HbA1C were determinants of CIMT in 331 subjects from a community-based prospective cohort study [49].

There was a significant negative correlation between plasma adiponectin and CIMT in patients with NAFLD. A significant inverse correlation was found between plasma adiponectin and CIMT in obese patients with NAFLD, but not in patients with NAFLD and T2DM. These results suggest that hypoadiponectinemia is much more relevant, as a cardiovascular risk indicator, in obese patients with NAFLD, with or without T2DM, but not in nonobese patients with NAFLD and T2DM. The significant independent predictors of the CIMT were diabetes duration, BMI, albumin/creatinine ratio, and serum cholesterol.

Fadaei et al. reported a negative a correlation between plasma adiponectin and CIMT in 49 Iranian patients with NAFLD [24]. De Almeida-Pititto et al. reported a significant inverse correlation between plasma adiponectin and CIMT in nondiabetic individuals with no cardiovascular disease in Brazil. However, they did not differentiate between obese and nonobese individuals, despite reporting that those with elevated CIMT had higher BMI and waist circumference [50]. Shargorodsky et al. reported an inverse relation between plasma adiponectin and CIMT in 47 obese nondiabetic individuals in Israel. However, they did not study this relationship in nonobese individuals or in patients with T2DM or NAFLD [51]. Tavridou et al. did not find any significant association between plasma adiponectin and CIMT in patients with T2DM with or without nephropathy [52]. In a previous work, our group reported a nonsignificant inverse correlation between plasma adiponectin levels and carotid IMT in nonobese Egyptian patients with T2DM. Multiple regression analysis revealed that plasma adiponectin was not a determinant of CIMT in that study group [10].

### 5.1. Limitations of the Study

Despite the important results of the study, we are aware of its limitations. The rather small number of the study group and the effect of dyslipidemia on CIMT, and lack of information about medications, particularly statins and aspirin, are major ones. The differences between the published research results might have been influenced by the different ethnic backgrounds of the participants.

### 5.2. Recommendations

A larger study to assess the relation between plasma adiponectin and CIMT in dyslipidemic and nondyslipidemic and obese and nonobese patients with NAFLD and T2DM will help to explore the antiatherogenic role of adiponectin in patients with NAFLD. These relationships may provide valuable data for understanding obesity pathophysiology and its role of in pathogenesis of NAFLD and other obesity-related complications and for the development of strategies to treat obesity and its complications and screen for risk of atherosclerotic cardiovascular disease among type 2 diabetic and nondiabetic obese patients. Different therapeutic approaches are targeted to increase adiponectin activities such as physical exercise, caloric restriction, administration of inducers as thiazolidinedione, omega 3, or recombinant adiponectin.

In conclusion, our results showed that plasma adiponectin is inversely correlated with CIMT in obese patients with NAFLD, but not in nonobese patients with NAFLD and T2DM. Hypoadiponectinemia could be a good indicator of cardiovascular risk in obese patients with NAFLD, with or without T2DM, but not in nonobese patients with NAFLD.

## Figures and Tables

**Table 1:** Demographic and laboratory data of the patients and control subjects.

Variable	Group I O+/NAFLD+/DM+ (*n* = 50) mean ± SD	Group II O-/NAFLD+/DM+ (*n* = 50) mean ± SD	Group III O+/NAFLD+/DM- (*n* = 50) mean ± SD	Group IV control (*n* = 50) mean ± SD	*P*value ANOVA	I*∗*II	I*∗*III	I*∗*IV	II*∗*III	II*∗*IV	III*∗*IV
Age (years)	54.3 ± 8.5	53.3 ± 8.4	46.7 ± 5.8	51.7 ± 9.5	<0.001	0.624	<0.001	0.084	<0.001	0.228	0.013
Diabetes duration (years)	11.3 ± 5.8	10.2 ± 5.8			0.253						
BMI (kg/m^2^)	35 ± 4	23.8 ± 1.2	37.1 ± 6.6	23.3 ± 1.4	<0.001	<0.001	0.266	<0.001	<0.001	0.101	<0.001
Fasting glucose (mg/dL)	192.4 ± 61.1	197.1 ± 69.5	94.5 ± 10.7	84.1 ± 5.7	<0.001	0.882	<0.001	<0.001	<0.001	<0.001	<0.001
2HPPG (mg/dL)	256.8 ± 77.2	266 ± 87.8	131.4 ± 19.1	120 ± 10.4	<0.001	0.825	<0.001	<0.001	<0.001	<0.001	0.005
HbA1c %	7.7 ± 1.2	7.5 ± 1	5.7 ± 0.5	4.6 ± 0.6	<0.001	0.679	<0.001	<0.001	<0.001	<0.001	<0.001
ALT (IU/L)	27.3 ± 15.7	27.5 ± 12.3	31.2 ± 19.2	22 ± 5.7	0.055	0.419	0.249	0.152	0.761	0.162	0.078
AST (IU/L)	26.3 ± 13	26.4 ± 8.7	31.1 ± 18	22.1 ± 5.3	0.019	0.348	0.075	0.242	0.317	0.035	0.003
GGT (U/L)	43.1 ± 25.1	41.5 ± 16.5	42.4 ± 19.1	38.8 ± 11.6	0.781	0.669	0.452	0.970	0.725	0.576	0.320
ALP (U/L)	132.2 ± 52.2	114.2 ± 43	115.9 ± 42.8	91.5 ± 29.7	<0.001	0.040	0.078	<0.001	0.785	0.003	0.001
ALB (mg/dL)	3.7 ± 0.5	3.6 ± 0.5	3.8 ± 0.3	4.1 ± 0.4	<0.001	0.531	0.073	<0.001	0.001	<0.001	<0.001
TP (mg/dL)	7.3 ± 0.4	7.2 ± 0.3	7.2 ± 0.3	7.2 ± 0.4	0.705	0.718	0.632	0.656	0.947	0.245	0.332
BIL T (mg/dL)	0.53 ± 0.21	0.52 ± 0.18	0.58 ± 0.2	0.46 ± 0.15	0.032	0.881	0.194	0.116	0.257	0.052	0.004
BIL D (mg/dL)	0.08 ± 0.08	0.07 ± 0.07	0.07 ± 0.06	0.04 ± 0.05	0.018	0.287	0.264	0.002	0.988	0.057	0.037
UREA (mg/dL)	27.6 ± 11.9	24.5 ± 8.1	24 ± 6.2	25.7 ± 9.1	0.587	0.270	0.253	0.663	0.937	0.435	0.472
Creatinine (mg/dL)	0.83 ± 0.28	0.78 ± 0.18	0.78 ± 0.16	0.75 ± 0.14	0.850	0.692	0.900	0.470	0.704	0.639	0.459
NA (m·mol/L)	139.3 ± 3.2	137.2 ± 2.9	138.1 ± 2.9	136.4 ± 2.9	<0.001	0.001	0.042	<0.001	0.174	0.087	0.002
K (m·mol/L)	4.2 ± 0.5	4.1 ± 0.4	4.1 ± 0.4	4.2 ± 0.5	0.740	0.328	0.474	0.841	0.787	0.388	0.675
UA (mg/dL)	5 ± 1.2	4.6 ± 1.1	5.7 ± 1.2	4.2 ± 0.6	<0.001	0.024	0.005	<0.001	<0.001	0.129	<0.001
Albumin/creatinine ratio	275 ± 295.7	214.4 ± 227.7	62 ± 50.5	21.9 ± 14.2	<0.001	0.244	<0.001	<0.001	<0.001	<0.001	<0.001
Cholesterol (mg/dL)	198.3 ± 45.5	173.6 ± 29.3	188.7 ± 33.8	155 ± 19.6	<0.001	0.001	0.260	<0.001	0.022	0.004	<0.001
TG (mg/dL)	192.4 ± 121.7	134.5 ± 81.8	149.9 ± 76.7	101.2 ± 21.9	<0.001	0.003	0.079	<0.001	0.161	0.255	0.001
LDL (mg/dL)	118.7 ± 38.3	103.9 ± 30.9	111.7 ± 35.4	91 ± 15.1	<0.001	0.035	0.370	<0.001	0.252	0.043	<0.001
HDL (mg/dL)	48.6 ± 15.5	49.8 ± 11.4	48.6 ± 10.9	46.1 ± 7.7	0.188	0.357	0.733	0.437	0.477	0.126	0.132
Plasma adiponectin (*µ*g/dL)	15.2 ± 3.1	26.3 ± 12.1	37.6 ± 20.5	47 ± 11.5	<0.001	<0.001	<0.001	<0.001	0.003	<0.001	0.020
CIMT (mm)	0.12 ± 0.03	0.11 ± 0.03	0.1 ± 0.02	0.07 ± 0.01	<0.001	0.008	<0.001	<0.001	0.094	<0.001	<0.001

The results are expressed as mean and standard deviation. O+/DM-/NAFLD+, obese patients with type 2 diabetes and NAFLD; O-/DM+/NAFLD+, nonobese patients with type 2 diabetes and NAFLD; O+/DM-/NAFLD+, obese nondiabetic patients with NAFLD; DM, diabetes mellitus; BMI, body mass index; FBG, fasting blood glucose; PPBG, postprandial blood glucose; HbA1c, glycated hemoglobin; UAC, urinary albumin creatinine ratio; TG, triglycerides; LDL, low-density lipoprotein; HDL, high-density lipoprotein; CIMT, carotid intima-media thickness. SD, standard deviation; ANOVA, analysis of variance; *P* < 0.05 is statistically significant.

**Table 2:** Comparison of CIMT and adiponectin regarding sex, obesity, and diabetes within the whole sample *n* = 200.

Variable	CIMT	*P* value	Adiponectin	*P* value
*Sex*				
Male	0.1 ± 0.03	0.643	31.2 ± 17.9	0.818
Female	0.1 ± 0.03		31.9 ± 17.8	

*BMI*				
Obese	0.11 ± 0.03	<0.001	26.4 ± 18.4	<0.001
Nonobese	0.09 ± 0.03		36.6 ± 15.7	

*DM*				
Diabetic	0.11 ± 0.03	<0.001	20.8 ± 10.4	<0.001
Nondiabetic	0.09 ± 0.02		42.3 ± 17.2	

BMI, body mass index; DM, diabetes mellitus.

**Table 3:** Correlation of CIMT and adiponectin with other parameters in patients with nonalcoholic liver disease.

Variable	CIMT		Plasma adiponectin	
	*r*	*P* value	*r*	*P* value
Plasma adiponectin (*µ*g/dL)	−0.458	<0.001		
Age (years)	0.132	0.062	−0.172	0.015
Diabetes duration (years)	0.306	0.002	−0.095	0.346
BMI (kg/m^2^)	0.351	<0.001	−0.297	<0.001
FBS (mg/dL)	0.592	<0.001	−0.546	<0.001
PPBG (mg/dL)	0.501	<0.001	−0.469	<0.001
HbA1c %	0.633	<0.001	−0.584	<0.001
Creatinine (mg/dL)	0.071	0.317	−0.01	0.893
Urine ACR	0.571	<0.001	−0.576	<0.001
Cholesterol (mg/dL)	0.364	<0.001	−0.208	0.003
TG (mg/dL)	0.288	<0.001	−0.147	0.038
LDL (mg/dL)	0.274	<0.001	−0.158	0.025
HDL (mg/dL)	−0.027	0.706	−0.034	0.628

CIMT, carotid intima-media thickness; BMI, body mass index; FBG, fasting blood glucose, PPBG, postprandial blood glucose; HbA1c, glycated hemoglobin, Urine ACR, urinary albumin creatinine ratio; TG, triglycerides; LDL, low-density lipoprotein; HDL, high-density lipoprotein; *r*, spearman correlation coefficient; *P* < 0.05 is statistically significant.

**Table 4:** Correlation between plasma adiponectin and CIMT in patients with nonalcoholic liver disease.

		Adiponectin	
		NAFLD + obese	NAFLD + T2DM
CIMT	*r*	−0.243	−0.032
	*P* value	0.015	0.753

CIMT, carotid intima-media thickness; NAFLD, nonalcoholic fatty liver disease; T2DM, type 2 diabetes mellitus; *r*, spearman correlation coefficient; *P* < 0.05 is statistically significant\.

**Table 5:** Predictors of CIMT in patients with nonalcoholic liver disease.

Sample size	200				
Coefficient of determination *R*^2^	0.473				
Multiple correlation coefficient	0.688				

Regression equation					
Independent variables	Beta coefficient	Std. error	*t*	*p* value	VIF

(Constant)	0.035	0.009	4.004	0.000	
Diabetes duration (years)	0.002	0.000	6.155	0.000	1.334
BMI (kg/m^2^)	0.00047	0.000	2.031	0.044	1.171
Albumin/creatinine ratio	0.00004	0.000	4.519	0.000	1.341
Cholesterol (mg/dL)	0.000	0.000	4.339	0.000	1.231

CIMT, carotid intima-media thickness; BMI, body mass index.

## Data Availability

The data used to support the findings of this study are available from the corresponding author upon reasonable request.
